# Chest CT findings in drug-resistant pulmonary tuberculosis: a comparative analysis of elderly and non-elderly patients

**DOI:** 10.1186/s12879-025-11766-w

**Published:** 2025-10-17

**Authors:** Guijuan Zhu, Weiguang Feng, Fang Li, Xin Zhang

**Affiliations:** 1 Department of Medical Imaging, Huai’an Infectious Disease Hospital, 128 Yan’an East Road, Qingjiangpu District, Huai’an, Jiangsu Province 223002 People’s Republic of China; 2 Department of Infectious Diseases, Huai’an Infectious Disease Hospital, Huai’an, Jiangsu People’s Republic of China

**Keywords:** Drug resistance, Pulmonary tuberculosis, Computer tomography, Imaging findings, Elderly patient

## Abstract

**Background:**

Drug-resistant pulmonary tuberculosis (DR-TB) remains a critical public health challenge, particularly affecting vulnerable populations such as the elderly, who exhibit higher morbidity and mortality rates. This study aims to elucidate the chest CT characteristics of DR-TB in elderly patients to improve diagnostic accuracy and guide individualized treatment strategies.

**Methods:**

A retrospective analysis of 183 confirmed DR-TB cases (Huai’an Infectious Disease Hospital, June 2013-June 2023) compared chest CT findings (lesion distribution, extent, and morphology) between elderly patients (≥ 60 years) and non-elderly patients (14–59 years).

**Results:**

Key findings reveal that elderly patients demonstrate a higher frequency of extensive lung involvement, with 76% exhibiting lesions in all lung lobes compared to 40.74% in the non-elderly group (*P* < 0.001). Additionally, the elderly group displayed significantly more pathological features, such as segmental and lobar shadows (61.33% vs. 45.37%, *P* = 0.033) and lung destruction (22.67% vs. 11.11%, *P* = 0.035).

**Conclusion:**

The identification of risk factors on chest CT, including the presence of pulmonary and bronchial lesions, highlights the necessity for tailored screening and management strategies for elderly DR-TB patients.

## Introduction

Drug-resistant pulmonary tuberculosis (DR-TB) remains a crucial public health challenge globally, significantly impacting morbidity and mortality rates, particularly among vulnerable populations such as the elderly [1]. The World Health Organization (WHO) has noted the alarming rise in drug-resistant strains of Mycobacterium tuberculosis, which complicates treatment regimens and leads to prolonged hospital stays and increased healthcare costs [2]. DR-TB, defined by resistance to at least isoniazid and rifampicin, presents a formidable challenge for tuberculosis prevention efforts due to its extreme infectivity, difficulty in achieving a cure, and significant mortality rate. The emergence of extensively drug-resistant strains further exacerbates these challenges, underscoring the urgent need for novel therapeutic strategies to effectively combat this public health threat [3, 4].

Despite advances in diagnostic techniques, including rapid molecular tests and improved imaging modalities, the timely identification of DR-TB remains a challenge, particularly in older adults who may present atypically due to age-related physiological changes [5]. Previous studies have highlighted age as a significant factor influencing the clinical presentation and outcomes of tuberculosis, raising concerns about the adequacy of existing treatment protocols for elderly patients [6]. Furthermore, the delay in diagnosis can lead to increased disease severity, necessitating a deeper understanding of the clinical and radiological features of DR-TB in different age groups [7, 8].

The existing literature presents a gap in comparative studies focusing on the clinical and radiological characteristics of DR-TB among elderly versus younger populations. While several studies have investigated the overall epidemiology of tuberculosis, there is limited research specifically addressing how age influences disease manifestation and treatment outcomes in DR-TB cases [9, 10]. This gap underscores the need for targeted investigations that can inform tailored therapeutic approaches and improve patient outcomes, particularly in the elderly demographic, which is often underrepresented in clinical research [11].

To address these gaps, this study conducts a retrospective analysis of clinical data spanning a decade from patients diagnosed with DR-TB, aiming to elucidate the distinctive chest CT findings in both elderly and non-elderly patients. By comparing the clinical and imaging characteristics of these two groups, we aspire to enhance diagnostic accuracy and inform treatment strategies tailored to age-related differences. The insights gained from this analysis will not only contribute to the growing body of literature on DR-TB but also support clinicians in making timely and effective management decisions, ultimately improving outcomes for affected individuals.

## Materials and methods

### Research subject

Between June 2013 and June 2023, a total of 14,337 patients underwent chest CT scans at Huai’an Infectious Disease Hospital. Among these, 221 patients were confirmed to have DR-TB via drug susceptibility testing.

Inclusion criteria were as follows: (a) positive sputum culture for Mycobacterium tuberculosis, (b) confirmed drug resistance via susceptibility testing, and (c) availability of comprehensive chest CT data. Exclusion criteria included: non-tuberculous mycobacterial lung disease, drug-resistant tuberculosis coexisting with HIV, ongoing radiotherapy or chemotherapy for malignant tumors, nephrotic syndrome, connective tissue diseases, liver and kidney transplantation, diabetes and other conditions that compromise immune function.

After applying inclusion and exclusion criteria, 183 patients were included in the final analysis. These patients were divided into two groups based on the age threshold of 60 years, as defined in China: the elderly DR-TB group and the non-elderly DR-TB group.

### Laboratory examination

Diagnostic criteria for pulmonary tuberculosis included a microscopically positive sputum smear, a positive Mycobacterium culture, and identification of the strain as part of the Mycobacterium tuberculosis complex group. The Roche culture medium and proportional drug susceptibility test were utilized to assess the drug sensitivity or resistance of the pathogenic bacteria, based on the observed colony growth.

### CT examination

Chest CT scans were conducted using a Philips Ingenity 64-slice spiral CT scanner. Patients were positioned supine, and scans were performed from the lung apex to below the costophrenic angle during breath-hold after deep inhalation. The scanning parameters included a tube voltage of 120 kV, automatic tube current modulation, with both slice thickness and spacing set at 3 mm. The pitch was 1.0, with a matrix size of 1024 × 1024.

All images were reviewed on the medical imaging PACS. Two deputy chief radiologists, specializing in chest diagnostics, collectively examined and documented the CT findings. In cases of disagreement, the images were further discussed with a senior physician to reach a consensus.

### Statistical analysis

Statistical analysis was conducted using IBM SPSS version 26.0 (SPSS, Chicago, IL). Continuous data were expressed as mean ± standard deviation and compared between groups using the t-test. Categorical data were presented as percentages and analyzed using the chi-square (χ^2^) test. A *P*-value of less than 0.05 was considered to indicate statistical significance.

## Results

### Demographic characteristics of the patients with DR-TB

There were no significant differences between the two groups in terms of gender distribution, treatment status or type of drug resistance (*P* > 0.05) (Table 1).

**Table 1:** The demographic features of elderly and non-elderly patients with DR-TB

Characteristics	Elderly group (*n* = 75)	Non-elderly group (*n* = 108)	χ^2^/*t*	*P*
Age	67.17 ± 5.94	38.71 ± 13.69	16.921	0.000
Gender			2.295	0.129
Male [n(%)]	59(78.67)	74(68.52)		
Female [n(%)]	16(21.33)	34(31.48)		
Treatment status			0.262	0.608
Initially treated [n(%)]	36(48.00)	56(51.85)		
Retreated [n(%)]	39(52.00)	52(48.15)		
Type of drug resistance			0.053	0.818
Primary	19(25.33)	29(26.85)		
Acquired	56(74.67)	79(73.15)		

### CT analysis of pulmonary lobe lesions in elderly and non-elderly patients with DR-TB

The proportion of lung lobe involvement in 1–2 lobes and 3–4 lobes among Elderly DR-TB patients was significantly lower than that among non-Elderly DR-TB patients (*P* < 0.05) (Table 2). Conversely, the proportion of whole-lung lobe involvement in Elderly DR-TB patients was significantly higher than that in non-Elderly patients (*P* < 0.05) (Table 2).

**Table 2:** Comparison of CT manifestations between elderly and non-elderly groups with DR-TB (total number of lung lobes involved)

Number of lung lobes involved	Elderly group (*n* = 75)	Non-elderly group (*n* = 108)	χ^2^	*P*
1–2 lung lobes [n (%)]	6 (8.00)	27 (25.00)	8.654	0.003
3–4 lung lobes [n (%)]	12 (16.00)	37 (34.26)	7.526	0.006
whole lung lobe [n(%)]	57 (76.00)	44 (40.74)	22.25	0.000

### Comparison of pulmonary lesions, tracheobronchial lesions, and pleural lesions on CT findings between elderly and non-elderly patients with DR-TB

**Fig. 1:**
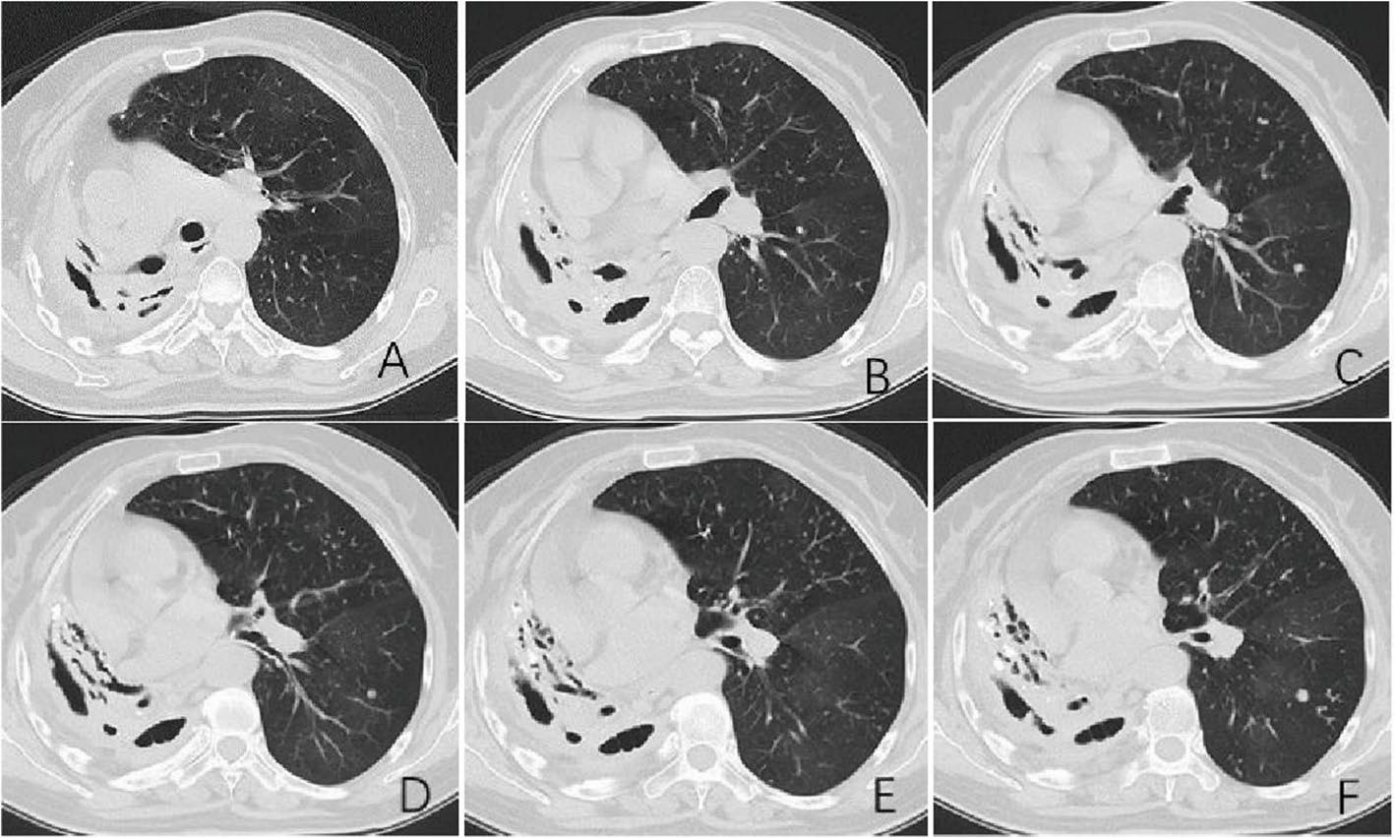
Representative chest CT findings in a 67-year-old female with DR-TB. Key features include: (i) destroyed right lung with segmental consolidation and air bronchograms; (ii) worm-eaten cavities and calcifications; and (iii) left-sided emphysema

**Fig. 2:**
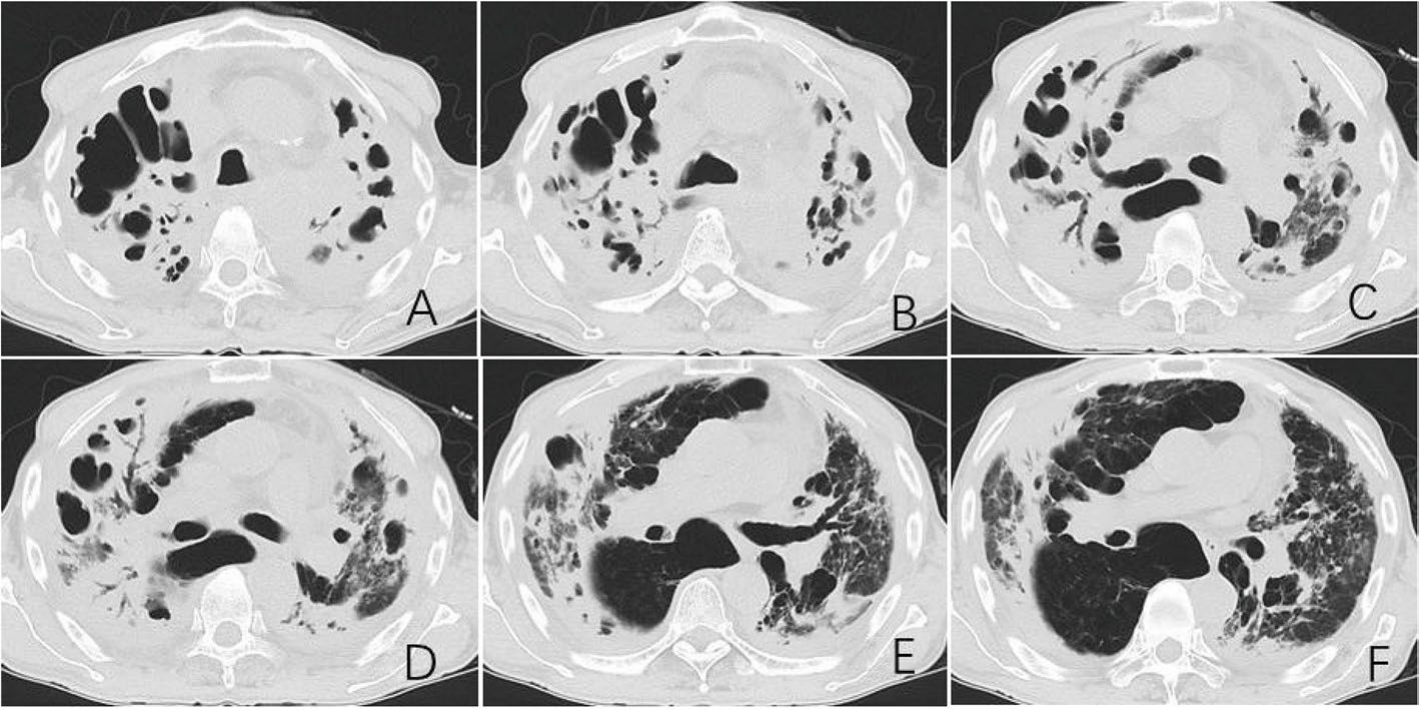
Representative chest CT findings in a 71-year-old male with DR-TB. Key features include: (i) bilateral lobar consolidations with air bronchograms; (ii) worm-eaten cavities and multiple cavities of varying sizes; and (iii) emphysema

**Fig. 3:**
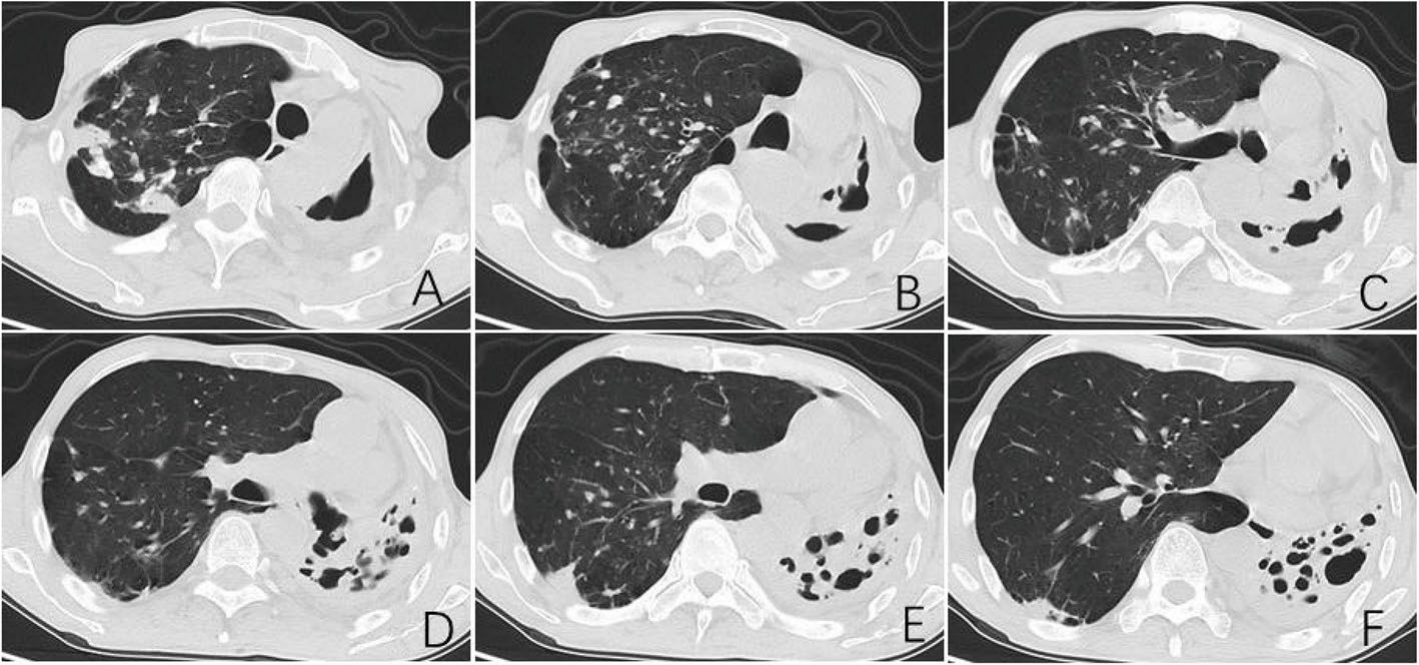
Representative chest CT findings in a 61-year-old male with DR-TB. Key features include: (i) destroyed left lung with segmental consolidation and air bronchograms; (ii) worm-eaten cavities; and (iii) right-sided emphysema

Among the pulmonary lesions, the incidence of segmental and lobular involvement, fibrous cords, calcifications, and destroyed lung in elderly patients with DR-TB was significantly higher than in non-elderly patients (*P* < 0.05) (Figs. 1, 2 and 3). Additionally, the incidence of small nodules/tree bud signs was significantly lower in elderly patients compared to non-elderly patients (*P* < 0.05). There was no statistically significant difference in the maximum lesion length, exudative patchy shadows, or proliferative nodules between the two groups (*P* > 0.05).

Regarding tracheobronchial lesions, the incidence of air bronchial signs and emphysema was significantly higher in elderly patients compared to non-elderly patients (*P* < 0.05). However, there were no significant differences in the incidence rates of bronchial stenosis/occlusion, bronchial wall thickening, traction bronchiectasis, varicose bronchiectasis, or pulmonary atelectasis between the two groups (*P* > 0.05). Furthermore, the incidence of bronchial dissemination was significantly lower in elderly patients compared to non-elderly patients (*P* < 0.05).

Among pleural lesions, there were no statistically significant differences in the incidence rates of free effusion, encapsulated effusion, pneumothorax, pleural thickening and calcification, or pleural nodules between the two groups (*P* > 0.05). Detailed comparisons are provided in Table 3.

**Table 3:** Comparison of Pulmonary lesions, tracheobronchial lesions, and pleural lesions on CT findings between elderly and non-elderly patients with DR-TB

CT findings		Elderly group (*n* = 75)	Non-elderly group (*n* = 108)	χ^2^/*t*	*P*
Pulmonary lesions	Segments and lobes [n(%)]	46(61.33%)	49 (45.37%)	4.518	0.033
	Maximum lesion length (mm)	69.77 ± 21.57	69.48 ± 19.05	0.096	0.924
	Exudative patchy shadow [n(%)]	55(73.33%)	86 (79.63%)	0.992	0.319
	Proliferative nodules [n(%)]	64(85.33%)	96 (88.89%)	0.509	0.475
	Fiber cord [n(%)]	52(69.33%)	53 (49.07%)	7.428	0.006
	Calcification [n(%)]	50(66.67%)	46(42.59%)	10.285	0.001
	Small nodule/tree bud sign [n(%)]	27(36.00%)	68(62.96%)	12.890	0.000
	Destroyed lung [n(%)]	17(22.67%)	12(11.11%)	4.431	0.035
Tracheobronchial lesions	Bronchial stenosis/occlusion [n(%)]	18(24.00%)	20(18.52%)	0.808	0.368
	Thickening of bronchial wall [n(%)]	19(25.33%)	21(19.44%)	0.898	0.343
	Tractive bronchiectasis [n(%)]	26(34.67%)	33(30.56%)	0.342	0.558
	Varicose bronchiectasis [n(%)]	20(26.67%)	17(15.74%)	3.275	0.070
	Disseminated lesions along the bronchus [n(%)]	27(36.00%)	68(62.96%)	12.890	0.000
	Air bronchial sign [n(%)]	45(60.00%)	46(42.59%)	5.365	0.020
	Emphysema [n(%)]	44(58.67%)	12(11.11%)	47.135	0.000
	Pulmonary atelectasis [n(%)]	16(21.33%)	13(12.04%)	2.868	0.090
Pleural lesions	Free effusion [n(%)]	4(5.33%)	3(2.78%)	0.785	0.375
	Encapsulated effusion [n(%)]	16(21.33%)	17(15.74%)	0.936	0.333
	Pneumothorax [n(%)]	2(2.67%)	1(0.93%)	0.831	0.361
	Pleural thickening and calcification [n(%)]	30(40.00%)	29(26.85%)	3.502	0.061
	Pleural nodules [n(%)]	5(6.67%)	7(6.48%)	0.002	0.960

### Comparison of mediastinal and hilar lymph node changes, pericardial involvement, and complications on CT imaging between elderly and non-elderly patients with DR-TB

There were no statistically significant differences in the incidence rates of mediastinal and hilar lymph node enlargement and calcification, lung volume reduction, mediastinal shift, and pericardial effusion between the two groups (*P* > 0.05). See Table 4.

**Table 4:** Comparison of mediastinal and hilar lymph node changes, pericardial involvement, and complications on CT imaging between elderly and non-elderly patients with DR-TB

CT manifestations		Elderly group (*n* = 75)	Non-elderly group (*n* = 108)	χ^2^	*P*
Mediastinal and hilar lymph node changes	Enlargement [n(%)]	11(14.67%)	11(10.19%)	0.840	0.359
	Calcification [n(%)]	2(2.67%)	4(3.70%)	0.150	0.698
Complications	Lung volume reduction [n(%)]	22(29.33%)	19(17.59%)	3.509	0.061
	Mediastinal shift [n(%)]	19(25.33%)	16(14.81%)	3.166	0.075
Pericardial disease	Pericardial effusion [n(%)]	7(9.33%)	7(6.48%)	0.509	0.475

### Comparison of CT manifestations of pulmonary cavitation between elderly and non-elderly patients with DR-TB

The incidence of worm-eaten cavities in the elderly drug-resistant tuberculosis group is higher than that in the non-elderly drug-resistant tuberculosis group, and the difference was statistically significant (*P* < 0.05) (see Fig. 1, 2 and 3). There was no statistically significant difference in the incidence rates of single cavities, multiple cavities, and the number of cavities Less than 3, number of cavities greater than or equal to 3, maximum cavity inner wall diameter, thin-walled cavity, thick-walled cavity, cavity wall thickness, cavity wall nodules, cavity air–liquid level, cavity wall calcification, cavity located in 1 lobe, cavity located in greater than or equal to 2 lobes, single lung cavity, bilateral lung cavity, cavity draining bronchus and satellite lesions around the cavity between the two groups (*P* > 0.05). See Table 5.

**Table 5:** Comparison of CT manifestations of pulmonary cavitation between elderly and non-elderly Patients with DR-TB

CT Manifestations of Pulmonary Cavitation	Elderly group (*n* = 75)	Non-elderly group (*n* = 108)	χ^2^/*t*	*P*
Worm-eaten cavities [n(%)]	42(56.00%)	43 (39.81%)	4.661	0.030
Single cavities [n(%)]	9(12.00%)	20(18.52%)	1.410	0.235
Multiple cavities [n(%)]	44(58.67%)	52(48.15%)	1.963	0.161
Number of cavities Less than 3 [n(%)]	12(16.00%)	18(16.67%)	0.014	0.904
Number of cavities greater than or equal to 3 [n(%)]	36(48%)	44(40.74%)	0.947	0.330
Maximum cavity inner wall diameter (mm)	32.57 ± 17.68	25.71 ± 18.49	1.853	0.065
Thin-walled cavity [n(%)]	18(24.00%)	26(24.07%)	0.000	0.990
Thick-walled cavity [n(%)]	38(50.67%)	58(53.70%)	0.163	0.685
Cavity wall thickness(mm)	6.76 ± 3.45	6.77 ± 3.53	−0.019	0.985
Cavity wall nodules [n(%)]	9(12.00%)	10(9.26%)	0.357	0.549
Cavity air–liquid level [n(%)]	4(5.33%)	6(5.56%)	0.004	0.948
Cavity wall calcification [n(%)]	5(6.67%)	4(3.70%)	0.830	0.361
Cavity located in 1 lobe [n(%)]	17(22.67%)	29(26.85%)	0.411	0.520
Cavity located in greater than or equal to 2 lobes [n(%)]	30(40.00%)	32(29.63%)	2.124	0.144
Single lung cavity [n(%)]	26(34.67%)	37(34.26%)	0.003	0.954
Bilateral lung cavity [n(%)]	24(32.00%)	29(26.85%)	0.570	0.450
Cavity draining bronchus [n(%)]	51(68.00%)	70(64.81%)	0.200	0.654
Satellite lesions around the cavity [n(%)]	50(66.67%)	72(66.67%)	0.000	1.000

## Discussion

In the present study, we systematically investigated the CT manifestations of DR-TB in elderly patients and identified distinct radiological features compared to non-elderly patients. Our findings revealed that elderly DR-TB patients exhibited more extensive lung involvement, with a higher prevalence of whole-lung consolidation, fibrous cords, calcifications, and damaged lungs (*P* < 0.01), as well as a greater incidence of air bronchial signs (60.00%) and worm-eaten cavities (56.00%) compared to their younger counterparts (*P* < 0.05). These observations underscore the unique phenotypic manifestations of DR-TB in the elderly, which are likely attributed to the prolonged disease course, recurrent infections, and diminished immune capacity [12].

The high incidence of calcifications in elderly DR-TB patients (*P* < 0.05) suggests a chronic infection course, as these deposits often result from the breakdown of local fatty acids and tissue necrosis, with calcium ions forming calcium phosphate or calcium carbonate deposits [13]. Furthermore, the coexistence of new and old lesions in elderly patients reflects the dynamic nature of the disease, where incomplete absorption, irregular treatment, and immune dysfunction contribute to repeated cycles of improvement and progression [14, 15]. This is further compounded by the reduced functionality of immune T and B cells in the elderly, which impairs the host's ability to effectively contain Mycobacterium tuberculosis, leading to unrestricted bacterial replication and the release of bacterial proteins [16, 17].

The association between emphysema and DR-TB in elderly patients (*P* < 0.01) highlights an important comorbidity that complicates disease management. Obstructive emphysema, often exacerbated by chronic inflammatory stimulation from tuberculosis, accelerates lung tissue damage and impairs drug penetration due to decreased blood supply [18]. This not only hinders effective treatment but also increases the likelihood of drug resistance, as poorly perfused lung tissue may fail to achieve therapeutic drug concentrations [19].

Deserving special attention is the higher prevalence of fibrous cords observed in elderly DR-TB patients. Fibrotic changes, as visualized on CT scans, are indicative of advanced tissue remodeling and may reflect the body's attempt to contain the infection through scarring and calcification [20]. However, this fibrotic process can also lead to irreversible structural changes, such as bronchiectasis and pulmonary varix, which perpetuate the cycle of inflammation and tissue damage [21]. The coexistence of fibrosis and active disease highlights the dual nature of DR-TB in elderly patients, where active infection and chronicRepair processes occur simultaneously, complicating both diagnosis and treatment [22].

Despite these significant findings, our study is not without limitations. The single-center design and relatively small sample size restrict the generalizability of our results, and the retrospective nature of the analysis introduces potential biases. Furthermore, not all patients underwent contrast-enhanced or high-resolution CT (HRCT) examinations, which were performed only based on clinical indications. Consequently, our comparative analysis was limited to non-contrast CT images, potentially restricting detailed evaluation of certain features such as vascularity, subtle parenchymal changes, or micronodular patterns. Additionally, the absence of comparative CT analyses across different types of DR-TB further limits the scope of our conclusions. Future studies should prioritize multicenter, prospective designs to validate these findings and explore the underlying mechanisms driving age-related differences in DR-TB presentations [23]. Moreover, the exploration of novel technical combinations integrating functional imaging and metabolic profiling could represent a promising avenue for delineating the pathological characteristics of DR-TB in elderly populations.

## Conclusion

This study elucidates the unique CT manifestations of DR-TB in elderly patients, emphasizing the importance of tailoring diagnostic and therapeutic strategies to address age-related variations. The distinct patterns of lung involvement and lesion characteristics identified in this study not only enhance our understanding of DR-TB but also provide valuable insights for improving clinical management and patient outcomes in this vulnerable population. Future research should continue to unravel the intricate relationships between age, immune dysfunction, and DR-TB progression, with the ultimate goal of developing targeted interventions to mitigate the burden of this devastating disease in elderly populations.

## Data Availability

The datasets generated and/or analyzed during the current study are available from the corresponding author on reasonable request.

## Abbreviations

CT: Computed tomography
DR-TB: Drug-resistant pulmonary tuberculosis
HIV: Human immunodeficiency virus
HRCT: High-resolution computed tomography
PACS: Picture archiving and communication system
WHO: World health organization
